# Genome Sequences of Seven Clade B2 Escherichia coli Strains Associated with Recurrent Urinary Tract Infections in Postmenopausal Women

**DOI:** 10.1128/mra.00035-23

**Published:** 2023-04-18

**Authors:** Jacob Hogins, Philippe E. Zimmern, Larry Reitzer

**Affiliations:** a Department of Biological Sciences, The University of Texas at Dallas, Richardson, Texas, USA; b Department of Urology, The University of Texas Southwestern School of Medicine, Dallas, Texas, USA; Loyola University Chicago

## Abstract

We report the genome sequences of seven recently isolated Escherichia coli strains from symptomatic postmenopausal women with a history of recurrent urinary tract infections. We have observed rapid laboratory evolution of strains after isolation. These strains were minimally passaged before analysis to prevent changes during culturing.

## ANNOUNCEMENT

Urinary tract infections (UTIs) are frequent bacterial infections, with Escherichia coli causing 80 to 90% of community-acquired UTIs (1). To better understand the molecular mechanisms underlying these pathogens, transcriptomic analyses are needed, as genomic studies have not identified UTI-specific virulence factors (2). However, analysis of RNA-sequencing data is facilitated by well-constructed genomes for comparison. Thus, to generate strong transcriptomic data, complete, high-quality genomes are preferred. We report the isolation and DNA sequencing of seven E. coli strains from women suffering from recurrent UTIs.

The isolation procedure minimized the potential for strain evolution caused by frequent serial passage on nonphysiologic media, such as lysogeny broth (LB) (3). Urine samples were collected based on an institutional review board (IRB)-approved study (approval number STU 032011-187 at The University of Texas Southwestern School of Medicine) in the clinic of Philippe E. Zimmern in Dallas, Texas. Bacterial cells were collected from clean-catch (voided) urine samples from symptomatic postmenopausal women with a history of recurrent UTIs who also had formal urinalysis results indicating nitrite positivity and moderate to large numbers of leukocytes. The ages of the women ranged from 49 to 78 years, with an average of 63 years. Two hundred microliters of urine was plated on chromogenic agar, and dark pink to purple single colonies, corresponding to E. coli, were selected for one final outgrowth in 5 mL LB. Cells were collected after 4 h of growth. Four milliliters was cryogenically frozen in LB with 20% glycerol, and 1 milliliters was used for phylogenetic typing as described (4). All strains were phylogenetic group B2, which is most frequently associated with UTIs (1).

For DNA isolation, cells from the frozen culture were scraped, inoculated into 2 mL of LB, and grown aerobically for 4 h at 37°C. The culture was split into two 1-mL fractions, pelleted by centrifugation, resuspended in phosphate-buffered saline (PBS) with lysozyme (25 mg/mL), and incubated for 1 h. The DNA was isolated following the protocol provided by New England Biolabs for Gram-negative bacteria via the Monarch genomic DNA purification kit. Once collected, DNA was submitted to SeqCenter (Pittsburgh, PA) for hybrid sequencing. The Illumina libraries were prepared using the Illumina DNA preparation kit and IDT 10-bp unique dual indices (UDI) and sequenced on an Illumina NextSeq 2000 system, producing 2 × 151-bp reads. Demultiplexing, quality control, and adapter trimming were performed with bcl-convert (v3.9.3) with default parameters (5). Sample libraries were prepared using the Oxford Nanopore Technologies (ONT) ligation sequencing kit (SQK-LSK109) with the NEBNext Companion Module (E7180L) in addition to native barcoding kits (EXP-NBD104 and EXP-NBD114), according to the manufacturer’s instructions. All samples were run with Nanopore R9.4.1 flow cells on a MinION Mk1B device. After sequencing, Guppy (v5.0.16) (6) was used for high-accuracy base calling (HAC) and demultiplexing with default parameters plus effbaf8; quality control and adapter trimming were performed with Porechop (v0.2.3) (7) with default parameters. Hybrid assembly with Illumina and ONT reads was performed with Unicycler (v0.4.8) (8), and assembly statistics were recorded with QUAST (v5.0.2) (9), both using default parameters. Unicycler produced circular closed genomes for those labeled complete and contigs for those labeled incomplete. No rotation was performed. Upon submission to NCBI, the genomes were annotated using PGAP (v6.3) with default parameters (10).

### Data availability.

The complete sequences were deposited in GenBank. The BioProject accession number is PRJNA909323. BioSample, SRA, and GenBank accession numbers are provided in Table 1.

**TABLE 1:** Relevant parameters for assembly and accession numbers for the strains described

Assembly	SRA accession no.	GenBank accession no.	Total no. of trimmed Nanopore reads	Total no. of raw Nanopore reads	Total no. of Illumina reads (R1 + R2)	No. of contigs	Largest contig size (bp)	Total length (bp)	Coverage of total genome (×)	Chromosome/plasmid	GC content (%)	*N*_50_ (bp)
KE21	SRS16265656	JAPVBX000000000	598,660	621,148	6,074,416	1	5,015,183	5,015,183	288	Chromosome	50.74	5,015,183
						1		113,382		Plasmid	51.04	
						1		15,472		Plasmid	41.13	
KE24	SRS16265657	JAPVBW000000000	416,818	432,011	6,424,926	5	4,871,255	4,911,042	270	Chromosome	50.60	4,871,255
						1		111,141		Plasmid	50.88	
						1		6,077		Plasmid	49.25	
KE25	SRS16265653	JAPVBV000000000	610,331	634,295	6,469,364	17	4,434,301	5,196,791	291	Chromosome	50.67	4,434,301
						1		143,143		Plasmid	51.9	
						1		5,630		Plasmid	47.39	
KE26	SRS16265654	JAPVBU000000000	1,482,284	1,529,140	6,077,780	1	4,910,688	4,910,688	414	Chromosome	50.68	4,910,688
						1		117,276		Plasmid	49.85	
						1		15,473		Plasmid	41.12	
						1		1,506		Plasmid	50.27	
KE4	SRS16265658	JAPVBT000000000	442,358	455,575	7,104,018	8	3,505,008	5,239,268	288	Chromosome	50.54	3,505,008
LRPF007	SRS16265659	JAPVBS000000000	658,819	680,447	5,804,374	33	2,589,798	5,056,897	285	Chromosome	50.43	2,589,798
						1		111,595		Plasmid	50.85	
LRPF62	SRS16265655	JAPVBR000000000	1,418,767	1,448,674	7,164,910	6	4,918,849	5,061,613	510	Chromosome	50.63	4,918,849
						1		148,249		Plasmid	51.91	
